# A Molecular Interpretation on the Different Penetration Enhancement Effect of Borneol and Menthol towards 5-Fluorouracil

**DOI:** 10.3390/ijms18122747

**Published:** 2017-12-18

**Authors:** Ran Wang, Zhimin Wu, Shufang Yang, Shujuan Guo, Xingxing Dai, Yanjiang Qiao, Xinyuan Shi

**Affiliations:** 1 Beijing University of Chinese Medicine, No. 11 of North 3rd Ring East Road, Chaoyang District, Beijing 100029, China; w13436525799@163.com (R.W.), WzMMzW@bucm.edu.cn (Z.W.); 18811795303@163.com (S.Y.); 18810820913@163.com (S.G.); jolly_1987@163.com (X.D.); 2 Key Laboratory of TCM-Information Engineering of State Administration of TCM, No. 11 of North 3rd Ring East Road, Chaoyang District, Beijing 100029, China

**Keywords:** penetration enhancement, molecular mechanism, coarse-grained molecular dynamics, menthol, borneol, 5-fluorouracil

## Abstract

Borneol and menthol are terpenes that are widely used as penetration enhancers in transdermal drug delivery. To explore their penetration-enhancement effects on hydrophilic drugs, 5-fluorouracil (5-FU) was selected as a model drug. An approach that combined in vitro permeation studies and coarse-grained molecular dynamics was used to investigate their penetration-enhancement effect on 5-FU. The results showed that although both borneol and menthol imparted penetration-enhancement effects on 5-FU, these differed in terms of their mechanism, which may account for the observed variations in penetration-enhancement effects. The main mechanism of action of menthol involves the disruption of the stratum corneum (SC) bilayer, whereas borneol involves multiple mechanisms, including the disruption of the SC bilayer, increasing the diffusion coefficient of 5-FU, and inducing the formation of transient pores. The findings of the present study improve our understanding of the molecular mechanism that is underlying 5-FU penetration-enhancement by borneol and menthol, which may be utilized in future investigations and applications.

## 1. Introduction

Transdermal drug delivery (TDD) has attracted considerable attention, particularly in terms of specific advantages, such as avoiding first-pass metabolism and improving patient compliance [1]. These features have rendered TDDs the appropriate method for the administration of 5-fluorouracil (5-FU). However, because of its hydrophilic properties, 5-FU does not readily permeate the stratum corneum (SC) barrier. Thus, 5-FU is commonly used as a model drug in the study of mechanisms to improve transdermal delivery [2,3]. Various methods of enhancing 5-FU permeation have been developed, and the co-administration with penetration enhancers (PEs) appears to be the most widely accepted technique, particularly when using natural products, such as terpenes [4,5].

Terpenes, which are naturally occurring volatile oils that are composed of hydrocarbons and their oxygenated derivatives, are the most widely accepted PEs based on their safety and high penetration-enhancing effect [6,7,8,9]. It has been suggested that these molecules enhance 5-FU penetration using two possible mechanisms; namely, complex formation and a form of facilitated transport. However, due to limitations in detection methods, a molecular explanation of the partitioning of 5-FU from the aqueous region of formulation into the hydrocarbon interior of the stratum corneum (SC) lipids remains elusive.

Molecular dynamics (MD) provides a convenient way of elucidating permeation processes and can yield important physical insights into related molecular mechanisms that could not be obtained from the usual diffusion cell experiments because of associated time and length scale [10]. Furthermore, larger temporal and spatial scales could be explored during simulation by altering the molecular resolution employing an approach using coarse-grained (CG) models [11,12]. Martini force field is the one of the common CG force fields that was developed by Marrink and his coworkers in 2007 [13], and has been reported to be a powerful method for the investigation of the bilayer system [14]. Thus, Martini CG MD can be expected to be a suitable approach for obtaining a molecular explanation of the 5-FU penetration-enhancement effect of terpenes.

Borneol and menthol are two terpenes that are extensively used as PEs [15,16,17,18]. In this study, the penetration-enhancement effects of borneol and menthol on 5-FU were investigated and compared using an approach that combined in vitro permeation studies and Martini CG MD. The results of the present study were thought to provide a molecular explanation of the partitioning of 5-FU from the aqueous region of formulation into the hydrocarbon interior of SC lipids.

## 2. Results and Discussion

### 2.1. In Vitro Permeation Studies on the Enahncement of 5-Fluorouracil (5-FU) Permeation by Borneol and Menthol

The rat skin can be used to gain the general insights into the transdermal pattern and acting mechanisms for penetration enhancers, though it is not a precise model for human skin for the percutaneous absorption [19,20,21].

To investigate the influence of PE concentration on the penetration enhancing effect, the following PE concentrations were assessed: 0.0%, 0.1%, 0.2%, 0.3%, 0.4%, 0.5%, 1%, 2%, and 3%. The results of various transdermal drug permeation parameters are presented in Table 1. When compared to the control group, both borneol and menthol showed a penetration enhancing effect on 5-FU at all of the PE concentrations studied, which followed a dose-dependent manner. However, when PE concentration was >0.5%, there was no additional penetration-enhancement. Such phenomenon has also been previously reported [22]. Another thing that needs to be noted was that because of lack of parallel experiments, we could not give the error range for ER, so the slight changes of ER at a PE concentration >0.5% will not be discussed here.

Comparative assessment of borneol and menthol indicated that borneol imparts a relatively stronger penetration-enhancement effect on 5-FU when compared to menthol. An earlier QSPR (Quantitative structure-property relationship) study on terpenes have revealed that hydrophobicity negatively influences the enhancement activity of terpenes on 5-FU [23]. The enhancement activity of borneol (logP = 2.71) was lower than menthol (logP = 3.2), which coincides with previous QSPR findings. However, when the logP values of borneol and menthol were included in a linear equation that was generated in the earlier QSPR study, the equation could not explain the observed difference between the two terpenes. Better results could always get by taking more factors into consideration, such as physiochemical descriptors [24] and aqueous pathway [25], but errors always exit. Therefore, in the present study, instead of focusing on the logP value and other physiochemical properties of borneol and menthol, we tried to attribute the detected differences to variations in penetration-enhancement mechanisms. The lipid-protein-partitioning (LPP) theory [26,27] describes two mechanisms that may be responsible for the observed differences in penetration-enhancement effects between borneol and menthol: 1. differences in their interaction with SC lipids; and, 2. differences in their effects on partitioning and diffusion of 5-FU.

### 2.2. Effects of Borneol and Menthol on Stratum Corneum (SC) Morphology Using Transmission Electron Microscopy (TEM)

**Figure 1:**
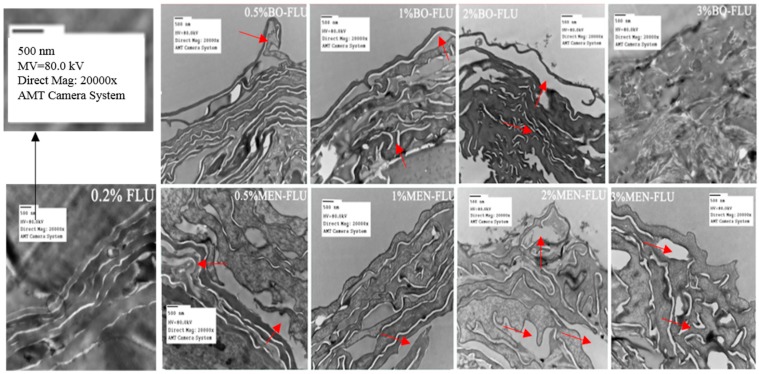
Transmission electron micrographs of the epidermis after 24 h of drug treatment (Magnification: 20,000 at 80 kV). From left to right, the concentration of PE was 0.0%, 0.5%, 1%, 2%, 3.0%. The picture labeled with “0.2% FLU” was the reference group not treated with penetration enhancer, 0.2% was the concentration of 5-FU, which was selected based on our pre-experiment, see Supplementary Materials. The picture labeled with “BO-FLU” was the group treated with borneol (**top**), and “MEN-FLU” for the group treated with menthol (**below**). The red arrows were used for the indication of disturbance on SC structure, and because of the obviously irreversible damage done by borneol at a penetration enhancers (PE) concentration of 3%, no arrows were used in the picture.

TEM was conducted to investigate the influence of borneol and menthol on SC morphology (Figure 1). When compared to the control group, the tightly packed lamellar structure was disrupted in with borneol and menthol treatment, and this effect was more distinct with an increasing PE concentration. Based on the observed changes in SC morphology, no differences in effect between borneol and menthol were observed at PE concentrations <2%. However, using 3% PE, the difference on the effect on SC morphology between borneol and menthol became distinct. In terms of the effect of 3% borneol on SC morphology, irreversible damage has been done to the skin. However, with menthol, the SC structure remained intact, although its disruptive effect became stronger as compared to that when using 2% PE. These findings indicate that both borneol and menthol disrupt SC morphology, and the differences in their effects could only be detected at a PE concentration of 3%, at which borneol showed stronger disruptive effects than menthol.

### 2.3. Effects of Borneol and Menthol on SC Morphology Using CG MD

The effects of borneol and menthol on SC morphology were also investigated using CG MD. Based on the work of Das and his coworkers in 2009 [28], a mixed ceramide lipid model was used in this assay, which is composed of a heterogeneous mixture of ceramides (CER), cholesterol (CHOL), and free fatty acids (FFAs) at a ratio of 2:2:1, simulating the bilayer, including its barrier properties and stability against mechanical stress. Furthermore, various concentrations of borneol and menthol were also used to investigate the influence of PE concentrations on the permeation enhancing effect. Because of differences in study scale between the in vitro permeation study and CG MD, PE concentrations may also vary.

**Figure 2:**
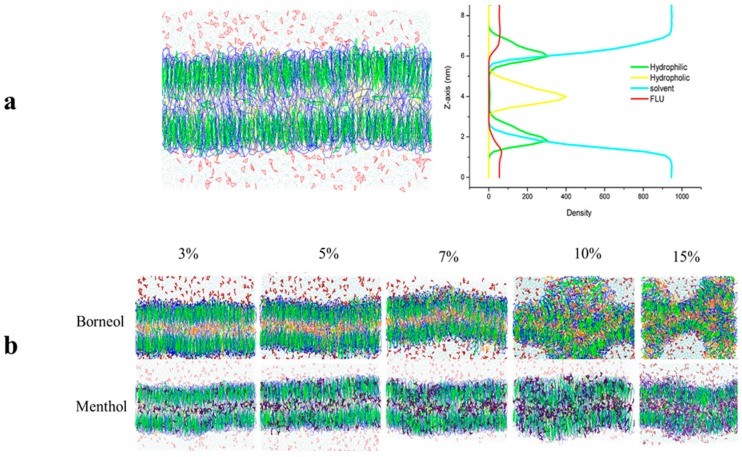
(**a**). The morphology of the initial bilayer together with its density distribution profile along the *z*-axis; (**b**). Stratum corneum (SC) bilayer morphology after exposure to different concentrations of borneol (**top**) and menthol (**below**).

The bilayer morphology after exposure to different concentrations of borneol and menthol was initially compared to obtain a general overview of their effect on the SC bilayer (Figure 2). When compared to the initial SC bilayer, both borneol and menthol could induce a slight curvature in the SC bilayer at PE concentrations below 7%, although no differences in effect were detected. However, at PE concentrations >10%, the borneol-treated bilayer showed more extensive disorganization, whereas that exposed to menthol remained intact, but exhibited greater curvature. These findings indicate that borneol imparts a stronger disruptive effect on the morphology of the SC bilayer at PE concentrations >10%.

**Figure 3:**
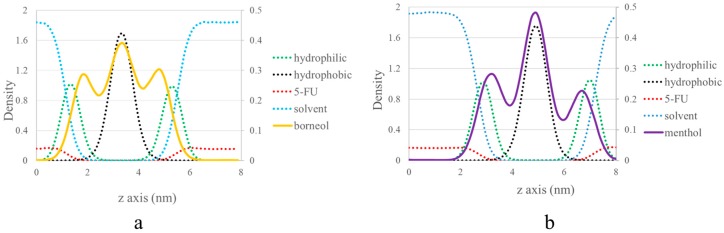
Corresponding density distribution profile of borneol (**a**) and menthol (**b**) along the *z*-axis at a concentration of 7% by coarse-grained (CG) molecular dynamics (MD). The other four components, including the hydrophilic group of ceramides (CER) (hydrophilic), the hydrophobic group of CER (hydrophobic), the solvent, and 5-FU, are displayed as dotted lines to depict the relative location of borneol and menthol, and their densities are indicated on the first axis on the left side. Because borneol and menthol show low-density distributions in the bilayer, their densities are depicted on the second axis on the right side.

Further investigations were carried out in CG MD to explore the mechanism that is underlying the observed differences in the effect of PEs on SC bilayer morphology was performed. Their relative position in the SC bilayer was evaluated by calculating their density distribution along the *z*-axis (the direction along the bilayer thickness). Figure 3 shows the effects of PEs at a concentration of 7%. To establish their relative position in the SC bilayer, the distribution of the four other components were also calculated, namely, the hydrophilic and hydrophobic groups of CER, the solvent, and 5-FU. These were plotted as a dotted line, and their density was indicated using the first axis on the left side of the density distribution curve. Because of the low-density distribution of borneol and menthol in the bilayer, their density was indicated using the second axis on the right side of the density distribution curve.

Three peaks in the density distribution curves of borneol (yellow) and menthol (purple) were detected. The highest peak was located at the same position as the hydrophobic group of CER, and the other two peaks were situated below the hydrophilic group of CER. Furthermore, borneol exhibited a lower-density distribution in the bilayer center and a higher-density distribution under the hydrophilic group of CER. Because borneol and menthol both contain a hydroxyl group, these interact with the hydrophilic group of CER through hydrogen bonding, which in turn influences the SC bilayer morphology [15,29]. Therefore, the higher distribution of borneol under the hydrophilic group of CER lipids might have resulted in stronger interactions with the hydrophilic group of CER, which may also be related to the stronger effect of borneol on the morphology of the SC bilayer at a PE concentration of >10% [30].

Our findings indicate that borneol and menthol interact with the hydrophilic group of CER, which in turn influences the bilayer structure. The higher-density distribution of borneol under the hydrophilic group of CER reveals that the interaction between borneol and the head group of SC lipids is stronger than that of menthol, thereby indicating that borneol has a stronger effect on the SC bilayer structure. When combining the results of our TEM experiment, the results in CG MD got on well with that in TEM experiment, both of them confirmed borneol’s stronger effect on SC bilayer structure. Therefore, in spite of their differences in differences in concentrations caused by different research scale, the two studies were related to each other from some extent.

### 2.4. The Effects of Borneol and Menthol on 5-FU Diffusion Using CG MD

**Figure 4:**
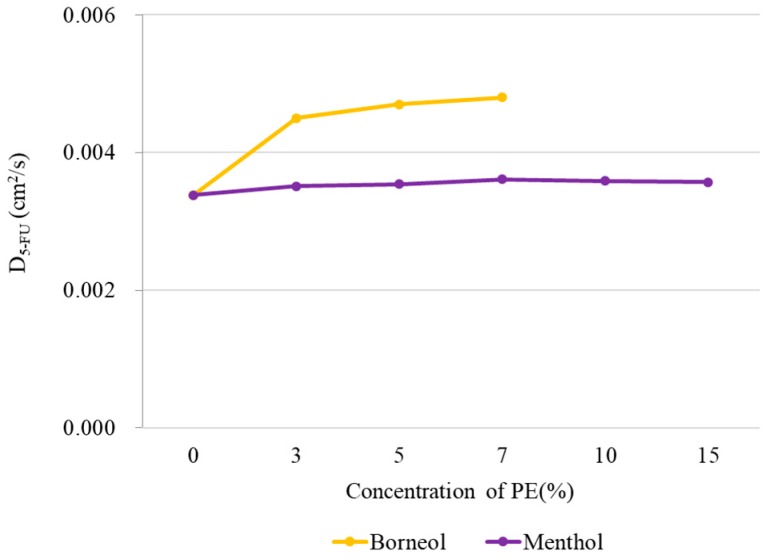
Diffusion coefficient of 5-FU along the *z*-axis at different PE concentrations obtained from CG MD. The application of high borneol concentration resulted in the disruption of membrane structure, so no meaningful diffusion coefficient for 5-FU could be calculated for borneol concentrations >10%.

To explore the influence of borneol and menthol on the diffusion of 5-FU, the diffusion constant of 5-FU along the axis of *z* (perpendicular to the bilayer surface) was calculated in CG MD using the Einstein relation:

(1) $$\Delta r{\left(t\right)}^{2}=\frac{1}{N_{5-FU}}\sum_{i=1}^{N_{5-FU}}{\left(r_{i}\;\left(t\right)-r_{i}\left(0\right)\right)}^{2}\;$$

(2) $$D_{5-FU}=\underset{t\to \infty }{\lim}\frac{1}{4t}\left(\Delta r{\left(t\right)}^{2}\right)$$

where $\Delta r{\left(t\right)}^{2}$ is the average mean square displacement of 5-FU molecular along the axis of *z* at time *t*. In this work, we employed 3000 configurations across 300 ns simulation time to perform the calculation over all 5-FU molecule (Figure 4). For menthol, no distinct changes in diffusion coefficient were observed with increasing PE concentrations, indicating that menthol imparts minimal effects on 5-FU diffusion. However, for borneol, the diffusion coefficient of 5-FU immediately increased in the presence of borneol, implying that borneol improves the permeation of 5-FU into the bilayer. Although the diffusion coefficient increased with higher PE concentrations, the rate of increase was minimal, suggesting that some other mechanism may be responsible for the stronger penetration-enhancement effect of borneol at concentrations >10%.

### 2.5. A Molecular Explanation of the Partitioning of 5-FU from the Aqueous Region into the Hydrocarbon Interior of SC Lipids

**Figure 5:**
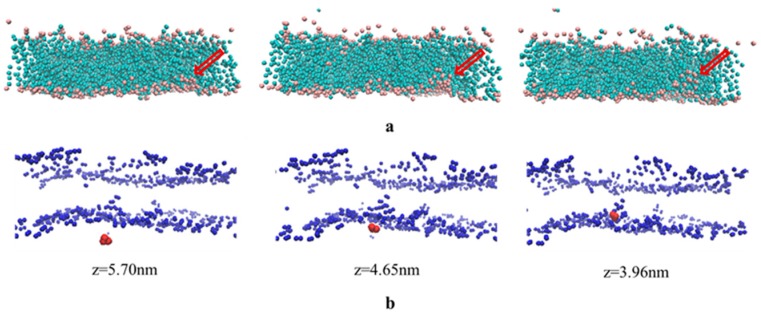
The observation of two process in CG MD, including transient pores formation process (**a**) and the diffusion process of 5-FU from the aqueous region into the hydrocarbon interior of stratum corneum lipids (**b**); (**a**) The aggregation of CER head groups in the bilayer center and formation of transient pores. The other components are set implicit, except for CER (pink dots for head groups and light green dots for tail groups), and the red arrow is used to indicate the position of the channel. (**b**) The diffusion of 5-FU from the aqueous region into the hydrocarbon interior of stratum corneum lipids aggregating around the CER molecule in the center of the bilayer, and the corresponding *z*-axis coordinates are shown underneath.

The findings of the present study allow for a deeper understanding of the mechanism of the penetration effect of borneol and menthol on 5-FU. However, our CG MD study was limited by PE concentrations, in that we did not investigate the penetration-enhancement effects at concentrations >10%. To address this component of our study and to investigate why borneol imparts a stronger penetration-enhancement effect at a PE concentration >10%, we used 5-FU as a model drug in our permeation study. Figure 5a shows that at a 15% borneol concentration, an increasing number of CER head groups was observed in the bilayer center (from left to right). Channels consisting of CER head groups were formed and were directly oriented toward the bilayer center. The hydrophilic property of the CER head groups suggests that the paths largely facilitate 5-FU permeation. Such paths have also been observed in an earlier study, which were described as transient pores [31].

The permeation of 5-FU molecules into the bilayer center was also observed in the molecular trajectory at a borneol concentration of 15%. Figure 5b shows that 5-FU molecules penetrate from outside the bilayer (*z* = 5.70 nm) to the center of the bilayer (*z* = 3.96 nm), with the help of transient pores.

**Figure 6:**
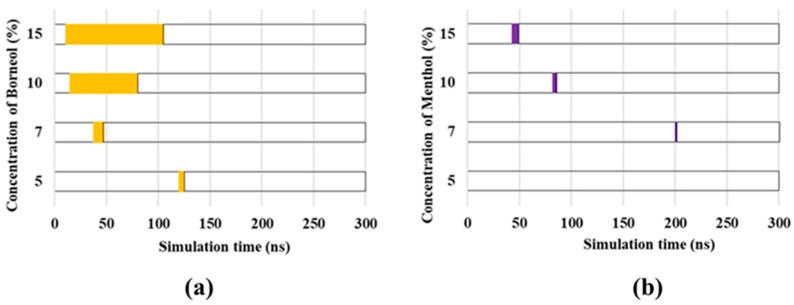
Molecular trajectory of borneol (**a**) and menthol (**b**) at different PE concentrations for 300 ns. Transient pore formation is depicted in purple and yellow, which, respectively, represents the time the transient pores were first observed and the time the transient pore disappeared.

Although the formation of transient pores was also observed in other groups, these substantially differ from each other, particularly in terms of the time that the transient pores emerged. When considering the importance of transient pores in 5-FU permeation, the duration of transient pore existence was used to evaluate the penetration-enhancement effect of borneol and menthol (Figure 6). The type and concentration of PEs largely influenced the time that transient pore was present. No transient pores were observed until PE concentrations were >3%. Then, with the increasing PE concentrations, more transient pores were formed, which in turn prolonged their existence within the SC bilayer. Furthermore, borneol-induced CER aggregates persisted for a longer period than menthol, particularly at PE concentrations >10%. The extended period for the opening of the transient pores may have largely facilitated in 5-FU permeation, which in turn results in a stronger penetration-enhancement effect on 5-FU at PE concentrations >10%.

## 3. Materials and Methods

### 3.1. In Vitro Permeation Studies

#### 3.1.1. Materials and Reagents

Borneol, menthol, and 5-FU (purity > 98%) were purchased from the National Institutes for Food and Drug Control (Beijing, China). Phosphate buffer saline (PBS, pH 7.2–7.4, 0.01 M) was purchased from Beijing Solarbio Science and Technology Co., Ltd. (Beijing, China). Glutaraldehyde (2.5%) was obtained from Biotopped Life Sciences (Beijing, China). Methanol (HPLC-grade) was supplied by Thermo Fisher Scientific (Beijing, China). 1,2-propanediol (PG), ether, and sodium chloride were purchased from Beijing Chemical Works (Beijing, China).

#### 3.1.2. Preparation of Samples

Terpene in a co-solvent system (20/80 (*v*/*v*) water/propanediol) was used for the delivery of 5-FU [32]. 5-FU was first dissolved in the co-solvent system at a final concentration of 0.2%. Then, the 5-FU solution was used as solvent to prepare different concentrations of borneol and menthol. The concentrations of borneol and menthol were 0.1%, 0.2%, 0.3%, 0.4%, 0.5%, 1.0%, 2.0%, and 3.0%.

#### 3.1.3. Preparation of Skin Samples

Skin samples were excised from male Sprague-Dawley rats (five weeks of age, body weight: 200 ± 10 g), which were supplied by Sibeifu Laboratory Animal Technology Co., Ltd., (Beijing, China). The rats were anesthetized with excess ether inhalation, and their abdominal skin was excised after removing hair with an animal hair clipper. After removing the fat and subcutaneous skin layers, the skin samples were washed with ultrapure water and 0.9% sodium chloride. All of the animal experimental procedures were conducted in conformity with institutional guidelines for the care and use of laboratory animals.

#### 3.1.4. Skin Permeation Assays

Freshly excised rat skin samples were immediately mounted on modified Franz-type vertical diffusion chambers. Each skin sample was sandwiched between the donor (upper) and receptor (lower) chambers, with the endothelial side in contact with the receptor chamber. The receptor chamber was filled with blank 80% propanediol (15 mL) and maintained at 32 °C with a constant magnetic stirring at the speed of 350 rpm. The donor chamber was filled with 2 mL of a mixed donor solution that was prepared as described in Section 3.1.2. The skin area for diffusion was 1.23 cm^2^. Samples of approximately 1.5 mL were collected from the receptor chamber and were replaced with an equal volume of blank 80% propanediol at the following time points: 2 h, 4 h, 6 h, 8 h, 10 h, 12 h, and 24 h. The solution samples were filtered through a 0.45-μm Millipore filter (Jin Teng, Beijing, China) and stored at 4 °C.

#### 3.1.5. Instrumentation and Chromatographic Conditions

The quantitative determination of 5-FU obtained in Franz diffusion cell experiment was performed with an HPLC system (Agilent 1100, Palo Alto, CA, USA) using methanol-water (5:95 *v/v*) in the mobile phase at a flow rate of 1.0 mL/min. The injection volume was 10 μl. A Waters Xbridge C18 column (250 × 4.6 mm, 5 μm, Waters, Milford, MA, USA) was used. The UV detector wavelength was set at 266 nm and the column temperature was maintained at 35 °C.

#### 3.1.6. Transmission Electron Microscope Studies

The skin samples were fixed instantaneously with 2.5% glutaraldehyde after permeation. Samples were then post-fixed in 1% OsO_4_ and were dehydrated in a graded series of acetone. The samples were subsequently embedded in a low-viscosity epon-epoxy mixture and sectioned. Thin sections were double stained with uranyl acetate and lead citrate, and then examined on a transmission electron microscope (JEOL JEM-1230, Tokyo, Japan) operated at 80 kV.

#### 3.1.7. Important Parameters

The main parameters used in this paper to assess the permeation enhancing effect were: the cumulative amount *Q_n_* (µg/cm^2^), the flux *J* (µg/cm^2^·h), and the enhancement ratio ER.

The quantity of drugs that permeated through SC is presented as cumulative amount *Q_n_* (µg/cm^2^) and is calculated using the following formula:

(3) $$Q_{n}=\frac{C_{n}\times V_{r}+\sum_{i=1}^{n-1}C_{i}\times V_{i}}{A}$$

where *C_n_* is the drug concentration of the receptor medium at each sampling time, *C_i_* is the drug concentration at *i*th sampling point, *V_r_* and *V_i_* were the volumes of receptor solutions and samplings, respectively, and *A* was the effective diffusion area of skin.

The zero-order permeating kinetics equation (*Q-t*) is obtained by regressing the cumulative amount on time:

(4) Q=Jt+BQ=Jt+B

where the slope *J* (µg/cm^2^·h) is the flux.

The permeability coefficient of drugs is related to flux using the following formula:

(5) $$K_{p}=J∕C_{0}$$

where *K_p_* (cm/h) is the permeability coefficient of drug, and *C*_0_ (µg/mL) is the initial concentration of drug.

The overall potency of PE is expressed as enhancement ratio (ER), a ratio of the *K_p_* value before and after enhancer treatment:

(6) $$\mathrm{ER}=K_{pe}∕K_{p}$$

where *K_pe_* is the *K_p_* value after treatment.

### 3.2. CG MD Simulation

#### 3.2.1. CG Molecular Models and Initial Structures

This assay mainly involved eight molecules, namely, 5-FU, borneol, menthol, CER, CHOL, FFA, PG, and water. The parameter files of the CG models of CER, CHOL, FFA, PG, and water are available in the Martini website, and the CG model of 5-FU, borneol, and menthol were developed according to the CG recipe published in the Martini website. The CG model of borneol and menthol were previously validated by our group [14,33]. For the CG model of 5-FU, the whole parameters and verification progress are presented in the Supplementary Materials.

**Figure 7:**
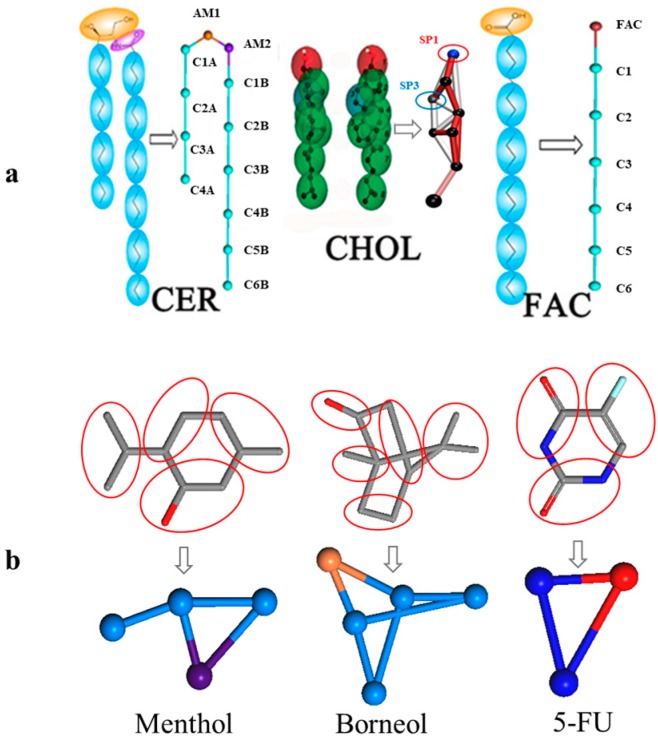
CG mapping of the six main compounds used in this study and the initial bilayer morphology. (**a**). CG mapping of the main bilayer components, including ceramides (CER), cholesterol (CHOL), and free fatty acids (FFAs), downloaded from the Martini website; (**b**). CG mapping of borneol (BO), menthol (MEN), and 5-fluorouracil (F-FU), which were built and optimized by our group.

The bilayer model of SC in this study was composed of CER, CHOL, and FFA at a 2:2:1 molar ratio, with properties previously validated by Das et al. [29]. The bilayer systems with different menthol concentrations in water were built using the Packmol package [34], and figures depicting lipid molecules were generated using Visual Molecular Dynamics (VMD) [35]. The CG model of the main molecules are shown in Figure 7.

#### 3.2.2. Simulation Details

Simulation was conducted with the GROningen MAchine for Chemical Simulation (GROMACS, ver. 4.6.3, University of Groningen, Groningen, The Netherlands). Prior to simulation, the system was relaxed through energy minimization (EM) using the steepest descent algorithm, through which the potential energy was descended to be negative on the order of 10^5^–10^6^, and the maximum force was adjusted to <80 kJ·mol^−1^. Standard simulation parameters that were associated with the MARTINI force field were used. The temperature was maintained using Berendsen Temperature, coupled with a time constant of 1.0 ps, and the pressure was controlled by a Berendsen Barostat and semi-isotropic pressure coupling with a constant of 3.0 ps and compressibility of 4.5 × 10^−4^/bar. The neighbor searching algorithm was implemented and the cut-off distance was set to 1.4 nm. The method was shifted and the cut-off length was picked at 1.2 nm for both the Van der Waals and electrostatic potentials. A time step was preset to 20 fs, and, finally, trajectory data for a total of 300 ns was gained. All of the parameters were selected based on the standard Martini simulation parameters.

## 4. Conclusions

The present study has shown evidence for the penetration-enhancement effect of borneol and menthol on 5-FU using in vitro permeation assays and CG MD. The results of CG MD provide a molecular understanding of the differences in the enhancement of 5-FU penetration between borneol and menthol. Menthol enhances 5-FU penetration by disrupting the SC bilayer, whereas borneol utilizes a more complicated mechanism, by which it increases its diffusion coefficient or forms transient pores. The existence of two penetration-enhancement mechanisms for borneol, thus renders it stronger in terms of its penetration-enhancement effects. The results of this study improve our understanding of the penetration-enhancement effects of borneol and menthol on hydrophilic drugs, which in turn may be utilized in future investigations and applications.

## Figures and Tables

**Table 1:** Permeation parameters of 5-fluorouracil (5-FU) across rat skin at various concentration of borneol and menthol.

PE Concentration	Borneol			Menthol		
	*J* (µg/cm^3^·h)	*Q*_24_ (µg/cm^3^)	ER	*J* (µg/cm^3^·h)	*Q*_24_ (µg/cm^3^)	ER
0.00%	0.82	20.45	1.00	-	-	-
0.10%	5.48	134.07	6.69	1.41	31.45	1.72
0.20%	6.40	160.36	7.80	2.13	46.95	2.60
0.30%	7.23	182.76	8.82	3.87	90.25	4.72
0.50%	12.51	299.43	15.26	3.85	96.94	4.70
1.00%	13.58	325.50	16.57	4.51	107.91	5.50
2.00%	14.19	342.11	17.31	4.05	96.71	4.94
3.00%	14.89	363.18	18.16	3.72	90.70	4.53

## Supplementary Materials

The following are available online at www.mdpi.com/1422-0067/18/12/2747/s1.
