# Supplementary material for: A Molecular Interpretation on the Different Penetration Enhancement Effect of Borneol and Menthol towards 5-Fluorouracil
Source: Int J Mol Sci. 2017 Dec 18;18(12):2747. doi: 10.3390/ijms18122747 (PMC5751346; doi:10.3390/ijms18122747)
Supplement: Supplementary file 1 [file ijms-18-02747-s001.pdf]

Supplementary Materials: A Molecular Interpretation on the Different Penetration Enhancement Effect of Borneol and Menthol towards 5-Fluorouracil

Ran Wang [1,2,†], Wu Zhimin [1,†] Shufang Yang [1,2], Shujuan Guo [1], Xingxing Dai [1,2], Yanjiang Qiao [1,2,*] and Xinyuan Shi [1,2,*]

1 Beijing University of Chinese Medicine, No.11 of North 3rd Ring East Road, Chaoyang District, Beijing 100029, China; wangran2017@sina.com (R.W.), WzMMzW@bucm.edu.cn (Z.W.), 18811795303@163.com (S.Y.), 18810820913@163.com (S.G.), jolly_1987@163.com (X.D.)
2 Key Laboratory of TCM-information Engineer of State Administration of TCM, No.11 of North 3rd Ring East Road, Chaoyang District, Beijing 100029, China
† These authors contributed equally to this study.
* Correspondence: yjqiao@263.net (Y.Q.); shixinyuan01@163.com (X.S.); +86-10-8473-8621 (X.S. & Y.Q.)

Academic Editor: name
Received: date; Accepted: date; Published: date

1. Parameterization and Optimization of the 5-FU CG Model

5-FU was one of the main molecular involved in this study, because no Martini force field parameter for 5-FU has been reported before, we parameterized it using method provided in Martini website. Their chemical structures and CG mappings are shown in **Figure S1a** and their force field parameters are shown in **Table S1**. Then in order to validate the accuracy of our model, we calculated the bond distributions of 5-FU in all atoms and CG levels, see **Figure S1c-S1e**. The distribution of CG was similar to that of all atoms, which confirmed the accuracy of our CG models.

Table S1. Force field parameters of 5-FU.

5-FU		
**Atoms** #	**Name**	**Type**
1	FU1	SP3
2	FU2	SP3
3	FU3	SNd
**Bonds** *i j*	**Length (nm)**	K_{bond} **(kJ·mol⁻¹·nm⁻²)**
1 2	0.21	20,000
1 3	0.24	20,000
2 3	0.10	20,000

What's more, since the oil-water partition coefficient was one of the important parameters in Martini force field, therefore, its oil-water partition coefficient was compared with the all atom model to confirm our CG model, see Figure Sb. Except for the good similarity between the all atom model and the CG model, 5-FU showed a high distribution in water than octanol, which was in consistent with the physiochemical property of 5-FU. All of this demonstrate that our CG model could be used for the further study 5-FU.

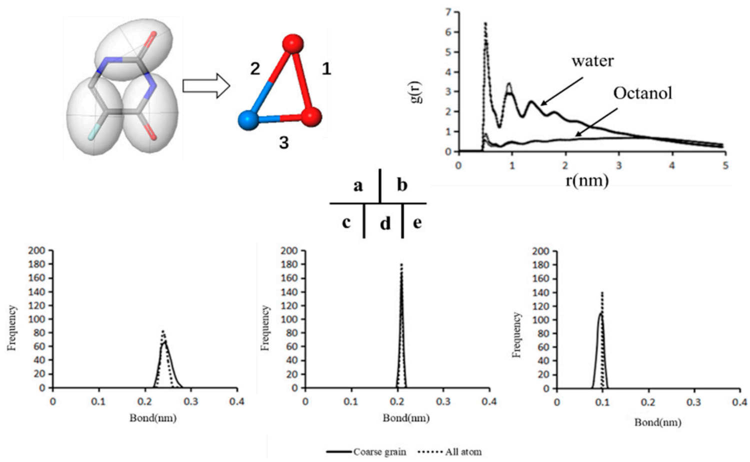

Figure S1. The CG mapping and validation of 5-FU CG model. a) 5-FU CG mapping. b) a comparison of 5-FU's oil-water partition coefficient between CG model (solid line) and all atom model (dotted line). c)-e) bond distribution frequency for bond 1-3.

2. Optimization of the 5-FU concentration in vitro permeation studies

Before comparing the penetration enhancement effect of borneol and menthol on 5-FU, pre-experiment has been done to study the proper concentration of 5-FU for vitro permeation studies. All the conditions used in this pre-experiment were the same as what we described in the manuscript. 6 different concentrations were investigated, including 0.1%, 0.2%, 0.3%, 0.4%, 0.5%, 0.8, and the Q-t curve was given in **Figure S2 a)**. As can be seen, the highest cumulative amount was given at a 5-FU concentration of 0.2%. What's more, the transmission electron micrographs of the epidermis treated by different 5-FU concentration was given in **Figure S2 b).** It was obviously that when the concentration of 5-FU was at 0.5% and 0.8%, the SC morphology of skin were largely disrupted, and this was thought to affect the penetration enhancement effect of PE. Therefore, considering the integral SC structure and high drug flux, 0.2% was select as the finial 5-FU centration for the latter study.

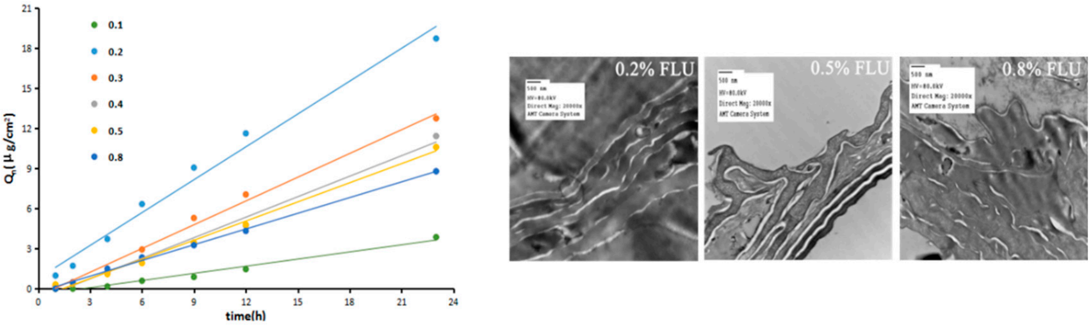

a) b)

Figure S2. Optimization of the 5-FU concentration in vitro permeation studies. a). The cumulative amount - time curve of 5-FU at different concentrations. b). Transmission electron micrographs of the epidermis after 24 h treatment of 5-FU at three different concentrations (Magnification: 20,000 at 80kV).
